# Creating Effective Mobile Phone Apps to Optimize Antiretroviral Therapy Adherence: Perspectives From Stimulant-Using HIV-Positive Men Who Have Sex With Men

**DOI:** 10.2196/mhealth.5287

**Published:** 2016-04-15

**Authors:** Keith J Horvath, Dawit Alemu, Thu Danh, Jason V Baker, Adam W Carrico

**Affiliations:** ^1^ Division of Epidemiology and Community Health School of Public Health University of Minnesota Minneapolis, MN United States; ^2^ Infectious Disease School of Medicine University of Minnesota and Hennepin County Medical Center Minneapolis, MN United States; ^3^ Community Health Systems School of Nursing University of California, San Francisco San Francisco, CA United States

**Keywords:** smartphone apps, technology adoption and use, men who have sex with men, stimulant drug use, HIV

## Abstract

**Background:**

The use of stimulant drugs among men who have sex with men (MSM) with human immunodeficiency virus (HIV) is associated with decreased odds of antiretroviral therapy (ART) adherence and elevated risk of forward HIV transmission. Advancing tailored and innovative mobile phone–based ART adherence app interventions for stimulant-using HIV-positive MSM requires greater understanding of their needs and preferences in this emerging area.

**Objective:**

The purpose of this study is to (1) assess reasons that stimulant-using HIV-positive MSM download and sustain their use of mobile phone apps in general, and (2) obtain feedback on features and functions that these men prefer in a mobile phone app to optimize their ART adherence.

**Methods:**

Focus groups were conducted with stimulant-using HIV-positive MSM (24-57 years of age; mostly non-Hispanic white; 42% once a week or more frequent stimulant drug use) in San Francisco and Minneapolis. Our aim was to explore the mobile phone app features and functions that they considered when deciding to download and sustain their use of general apps over time, as well as specific features and functions that they would like to see incorporated into an ART adherence mobile app. Focus groups were audiorecorded and transcribed verbatim. Thematic analysis was applied to transcripts using line-by-line open coding and organizing codes into meaningful themes.

**Results:**

Men reported that they currently had a variety of health and wellness, social media and networking, gaming and entertainment, and utility apps on their mobile phones. Downloading apps to their mobile phones was influenced by the cost of the app, recommendations by a trusted source, and the time it takes to download. In addition, downloading and sustained use of apps was more likely to occur when men had control over most features of the app and apps were perceived to be useful, engaging, secure, and credible. Participants suggested that ART adherence mobile phone apps include social networking features, connections to local resources and their medical chart, and breaking HIV news and updates. Although some men expressed concerns about daily self-monitoring of HIV medication doses, many appreciated receiving a summary of their medication adherence over time and suggested that feedback about missed doses be delivered in an encouraging and humorous manner.

**Conclusions:**

In this study, we were able to recruit a relatively high proportion (42%) of HIV-positive MSM reporting weekly or more stimulant use. These results suggest critical design elements that may need to be considered during development of ART adherence-related mobile phone apps for this, and possibly other, high-risk groups. In particular, finding the optimal balance of security, engagement, usefulness, control capabilities, and credibility will be critical to sustained used of HIV treatment apps.

## Introduction

Men who have sex with men (MSM) continue to bear the heaviest burden of new human immunodeficiency virus (HIV) infections in the United States [1]. Studies show that illicit substance use remains high among MSM [2,3], with approximately half reporting non-injection substance use in the past year [4]. Among HIV-positive persons, the use of stimulants (eg, methamphetamine) is associated with decreased odds of antiretroviral therapy (ART) utilization, difficulties with ART adherence and persistence, elevated HIV viral load, and elevated risk of forward HIV transmission [5-12]. Optimizing HIV disease management reduces excess morbidity and mortality among persons with HIV [13] and lowers the probability of forward transmission to uninfected sexual partners [14]. However, it is estimated that only 27% of MSM with HIV are virally suppressed [15,16]. Therefore, advancing tailored and innovative ART adherence interventions for stimulant-using HIV-positive MSM remains a high priority [17].

The use of technology to address the HIV prevention and care needs of persons most at risk for infection and poor treatment outcomes has increased in recent years [18,19]. A recent review of technology-based interventions addressing the HIV prevention and care continuum found 18 current or in-development mobile phone-based intervention studies [20]. However, the efficacy and effectiveness of mobile phone-based intervention approaches is still largely unknown, and understanding of target populations’ technology access and use is critical for advancing work in this area [20]. A qualitative study by Goldenberg et al of HIV-negative MSM’s preferences for a mobile HIV prevention app showed that men favored apps that addressed multiple health issues, allowed them to connect socially with other men, and were credible, customizable to their needs, simple and easy to use, interactive, and secure [21]. Schnall et al [22] conducted focus groups of HIV-positive persons (74% male; mostly racial and ethnic minority; and 54% mobile phone users) and used self-determination theory [23] and Fogg’s functional role triad [24] to assess how mobile apps may be used to meet the health care needs of people with HIV. Participants in this study suggested use of mobile phone calendars and reminders, self-monitoring of lab results, use of mobile phones for providers to reach out to patients for education and for video lectures, games, and rewards, the use of mapping systems to locate support groups, and ways to network with other people with HIV [22].

These recent studies on mobile phone app preferences among HIV-negative MSM and HIV-positive men and women provide insights into best practices for mobile phone apps for these populations. However, it is unknown whether these same preferences are held by stimulant-using HIV-positive MSM, who may have different technology-assisted prevention and treatment needs than their HIV-negative or non-stimulant-using counterparts [25]. Therefore, we conducted focus groups with stimulant-using HIV-positive MSM to address two primary research questions: (1) What features and functions of mobile phone apps does this population consider when deciding to download and sustain their use of apps over time, and (2) What features or functions do these men prefer in a mobile phone app to help them manage ART adherence?

## Methods

### Participants

Five focus groups, two in San Francisco and three in Minneapolis, were conducted in April-June 2014. Participants for the focus groups were recruited through local acquired immune deficiency syndrome (AIDS) service organizations, substance abuse treatment centers, and word of mouth. A total of 26 participants (16 from San Francisco and 10 from Minneapolis) participated in the study.

Inclusion criteria for the study were self-reported: (1) male 18 years of age or older, (2) having had sex with another man in the past 5 years, (3) stimulant use (methamphetamine, cocaine, crack cocaine, amphetamine and/or ecstasy) in the past 6 months, (4) diagnosis of HIV and taking ART, (5) having access to or owning a mobile phone with mobile phone features, and (6) English speaking.

### Procedures

All procedures were approved by the University of Minnesota Institutional Review Board. Focus group questions were developed by the research team and guided by the Technology Adoption Model (TAM) [26]. The TAM is a conceptual model to capture how persons will come to accept and use a new technology, such as the perceived usefulness of the technology and how easy the technology is to navigate. Participants were asked to describe what apps they currently have on their mobile phone. Next, men were asked to reflect on features and functions of mobile phone apps in general that they believed contributed to their decision to download, initiate use of, and continue to use apps. Although focus group members often spontaneously mentioned many features (eg, perceived usefulness) of their general app downloading and use that were relevant to the TAM conceptual model, we probed men to reflect on factors of the TAM conceptual model that were not mentioned. Finally, men were also asked to describe features and functions that they would like to see in ART adherence apps.

Recruitment materials included a link to the study website where interested persons were welcomed and asked to complete eligibility screener items, from which sociodemographic and drug use data in Table 1 were obtained. Those who met eligibility criteria were asked to provide consent. Focus groups were conducted in confidential settings in both locations. To maintain confidentiality and promote truthful answers to socially sensitive questions, participants were encouraged to use and refer to each other by their first name only. Focus group discussions lasted from 90-105 minutes. All focus groups were digitally recorded and later transcribed for analysis.

### Data Analysis

Thematic analysis was used to analyze transcripts [27]. Qualitative codes were developed using line-by-line open coding for all transcripts [28], with the unit of analysis being a complete thought reflecting one of the codes. As such, the unit of analysis could range from several words to multiple sentences. First, 2 authors (DA and TD) coded one transcript to individually identify preliminary themes (DA and TD). The authors met to discuss their themes and refine the coding scheme. Once codes were agreed upon, the remaining transcripts were coded. Disagreements were resolved with discussion [29]. Finally, 3 authors met (KH, DA, and TD) to debrief about the study findings and organize the themes within meaningful categories (as described below).

## Results

Participants were 24-57 years of age, with an average of 43 for men in San Francisco and 40 for those in Minneapolis. Most men were white (25/26, 96%) and non-Hispanic (23/26, 88%), and the average self-reported ART adherence in the past 2 months was 87%. There was a variation in frequency of stimulant drug, with 42% of men reporting stimulant drug use at least once a week or more frequently (see Table 1).

Table 2 shows the apps that men in the focus groups reported currently having on their mobile phone. Overall, men reported downloading and currently using a variety of health and wellness, social networking and dating, gaming and entertainment, and utility apps. Of relevance to stimulant-using HIV-positive MSM, focus group members reported currently having apps to assist them with maintaining their sobriety, clinical care, insurance, pharmacy, medications, and HIV.

The study team organized themes that emerged from the focus groups into three main categories: reasons men downloaded apps to their phones, general app design features that convinced men to download and sustain use of apps over time, and preferences for components and features of an ART adherence app. The organization of the themes and definitions are shown in Table 3.

**Table 1:** Focus group sociodemographic characteristics.

		Total, N=26	San Francisco, n=16	Minneapolis, n=10
Age (in years), mean		41	43	40
Race, col%^a^				
	White	96	94	100
	African American	4	6	0
Ethnicity, col%^a^				
	Hispanic	12	13	10
	Non-Hispanic	88	87	90
ART adherence, %	Self-reported in past 2 months^b^	87	87	86
Stimulant drug use, past 6 months, col%^a^				
	Less than once a month	23	6	50
	Once a month	23	30	10
	Every couple of weeks	12	12	10
	Once a week	22	25	20
	2-5 days a week	8	12	0
	Almost every day or every day	12	12	10

^a^Column percentage.

^b^ART adherence was assessed with the following item: “What percent of your prescribed HIV medication have you taken in the last 60 days (or about the last 2 months)? This may not be 100% for many people (eg, 0% means you have taken no medication, 50% means you have taken half your medication, 100% means you have taken every single dose of medication and at your usual scheduled time). If you’re unsure, make a best guess” with a pulldown menu of percentages from 0-100% with response options at 1% increments.

**Table 2:** Types of apps downloaded by participants.

App types		Examples
**Health/wellness apps**		
	General/other health	WebMD, Sobriety Day Counter, First Aid, Run Keeper
	Clinic/Insurance/Pharmacy	Kaiser Permanente, MyChart, Blue Cross/Blue Shield, Walgreens
	Medication	Pill Finder, Prescription Tracker
	HIV	HIV Plus
**Social media/networking apps**		
	Social networking	Snapchat, Facebook, Twitter, Skype, YouTube, TubeMe, WhatsApp, Viber
	Dating/sex seeking	Grindr, Scruff, Growlr
**Gaming/entertainment apps**		
	Movies, TV, and photos	Netflix, Flickr, Flickster
	Music	Spotify
	Gaming	Candy Crush Saga
**Utility apps**		
	General/Other	Google Maps, Smart Ride, Google Chrome, Dropbox, Calendar, Dual Lingo, Yelp, Photoshop
	Banking	Wells Fargo, TCF Banking

**Table 3:** Themes and definitions.

Theme		Definition
**Reasons to download an app**		
	Cost	How much is acceptable for an app to cost to consider downloading an app
	Recommendations from friends	Downloading an app was influenced by recommendations from friends
	Time to download	The time it takes to download influenced men’s decisions to download the app to their phone
**App design features associated with downloading** *and* **sustained use**		
	Control	Beliefs that having control over the different features/functions of the app is important
	Perceived usefulness	Beliefs that an app makes their life easier or is useful in their lives
	Engaging	Statements that an app has to be visually engaging, fun, and/or keep them interested
	Credible	Beliefs that the app has to be credible or come from a credible source
	Security/Privacy	Statements about security and how that might influence downloading or using an app
	Ease of use	Perception that the app is simple or easy to use or simple to navigate
**Preferences for ART adherence app features and functionality**		
	Social networking	Beliefs that being able to network with other HIV positive people would be useful
	Resources	Statements that using the app to connect with local resources to help them manage their HIV or other nearby community resources would be helpful
	HIV news/updates	Having updated HIV information would be useful
	Synching capabilities	Beliefs that synching the app to other apps on their mobile phone/computer or synching with their electronic medical record would be helpful
	Reminders for health care	Having a reminder for their medical appointment and other medical needs is important
	Self-monitoring	Perceptions of how medication reminders should operate and specific ideas for medication reminder system
	Adherence performance feedback	Statements about how feedback about their ART adherence performance or other aspects of their life (eg, diet) should be presented
	Graphing and summarizing	Participants’ desire to have a feature that would graph their ART adherence performance or summarize their health over time
	Health file	Men’s beliefs that having a place to store their health information would be valuable

### Reasons to Download an App

Participants identified three reasons that contributed to downloading an app to their phone.

#### Cost

Most men preferred a free app. However, some men did not mind paying a modest amount for an app so long as they heard about the product from a trusted source: “I might pay for an app if I know that it’s really good and someone has recommended it but I’m certainly going to try the free version first” (41, non-Hispanic white, stimulant use once a week).

#### Recommendation From Friends

A recommendation from a friend was key in men’s decisions to download an app: “Word of mouth, like if someone says, ‘Oh this is a great app,’ then I’m probably going to use that then too” (44, non-Hispanic white, stimulant use less than once a month).

#### Time to Download

The time it takes to download an app was another key consideration in their decision to download an app. Most men favored an app that takes little time to upload or update. As one respondent noted: “you sit there and wait, you know, forever for it to upload and, you know, somebody’s standing right next to you. He’s on a different app, and there’s his immediate” (49, non-Hispanic white, stimulant use almost every day or every day).

### Design Features and Functions Associated With App Downloading and Sustained Use

Participants identified six general design features of apps that they used to determine whether they would download an app to their phone and continue to use it over time.

#### Control

Overall, men were comfortable with most features of an app as long as they had control over it: “all of the features should really be under the control of the user…very customizable. This is I think what…what keeps me coming back, knowing that I can adjust” (48, non-Hispanic white, stimulant use almost every day or everyday). Some of the features men want to have control over include setting up alarm features, getting email reminders, and push notifications:

When you got it registered, ask more push notifications, then that just kind of makes it simple for…for everybody. I mean, you know, if you don’t like push notifications, you can…check that box. If you do like push notifications, you can. And it…makes it available for those who do like it…But it is not a hassle for those who don’t, at the same time. I think that would just make it easy.

27, African-American, stimulant use less than once a month

#### Perceived Usefulness

Participants believed that apps should be something that they can integrate into their daily lives, and they may delete apps that are not functional:

For me, um, you know, apps is usually, uh, it’s, it’s gonna be functional...if it seems relevant or could be helpful, you know, I’ll just get, get one to, to browse. And then the thing that I do is to explore. What I do is I, uh, I don’t have apps I don’t use on my phone. I get rid of them.

42, non-Hispanic white, stimulant use every couple of weeks

Men stated that the app should be something that enhances their life and takes care of their basic needs. As one participant stated, “It definitely is practical. You know, is it working in my life? How can it enhance my life? Not just, you know, just to have on the phone” (37, non-Hispanic white, stimulant use almost every day or every day).

#### Engaging

Overall, men would like to see visually engaging apps that use bright colors and interactive features: “It has to be really fun and like, interactive and you know, it has bright colors and you know, that’s a good thing” (27, African-American, stimulant use less than once a month). Most men agreed that an app that was not engaging would be reason for discontinuing its use: “if it looks like a spreadsheet…I’m not gonna use it. You know…or if it’s just too clinical, you know, as to the color and…the design and style” (46, Hispanic white, stimulant use once a week).

#### Credible

Nearly all of the participants agreed that the app should come from a trusted source. Some mentioned universities as examples of possible trusted sources of the future app to be developed. They also mentioned that the source should be displayed for the users to easily see:

I think um, knowing the um, the source of the app is pretty important, like, credible, reliable you know, sources right up front so that…you know, it’s like university would be, obviously, credible, you know. They wouldn’t be any doubt.

42, non-Hispanic white, stimulant use once a month

#### Security/Privacy

There was some variation in men’s concern with regard to privacy, with some men expressing little concern about security while using apps, while others were reluctant to disclose too much personal information over concerns about security:

So I found out about this HIV Plus and installed it, and it really wanted to know everything about me possible. And I thought, my god, I’m not entering all that information on an app on my telephone. You know, I’m just not gonna do it. And so I never used it. Ever

41, non-Hispanic white, stimulant use less than once a month

Others were comfortable sharing their personal information as long as they are informed about the need for the information. As one participated stated, “As long as it’s clear as to what …what it wants to access and why…I’m usually okay” (46, non-Hispanic white, stimulant use less than once a month).

Men preferred a tight security system on the app and suggested several layers of security to be put in place, especially using more than one password, to safeguard their account. However, participants also mentioned that they do not want to be asked to change their online security information frequently, as is required by some apps:

And so I would want to make sure that there is going to be adequate protection…so I would consider a couple layers, I mean, definitely, more than one password, um, but I don’t want constantly changing security questions every three months and on and on and on that some of the sites do.

54, non-Hispanic white, stimulant use every couple of weeks

#### Ease of Use

Overall, men wanted an app that was simple to use: “it needs to be easy…the more complicated you make it, the less easy it is” (41, non-Hispanic white, stimulant use less than once a month). Consistent with this, participants preferred infrequent changes in an app since learning new features is time consuming:

What I really hate is when the app changes the configuration and you go to it and it looks like a completely different thing and uh, because of an update. And you have to go and you to relearn this whole process to get to what you want to get at. And that’s annoying. And…and especially how often they have updates and changes. And it’s like, “Okay. That wasn’t necessary. And now, it’s just a bunch of work.” And…and the more it changes they have and the more work it is, the less I’m opt to use it.

42, Non-Hispanic white, stimulant use once a month

### Preferences for Antiretroviral Therapy Adherence App Features and Functionality

Nine themes related to men’s preferences for features and functionality of an ART adherence app emerged from the focus groups, which are described below.

#### Social Networking

Nearly all men expressed their desire to connect with other people having the same HIV status. Some preferred a dating site where they can meet other men for sex in a confidential manner:

To being able to…reach out to…people who have HIV in your community. Um, I know that a lot of apps like Grindr and more sexual hookup type of sites that are probably end up happening on that as well. It doesn’t have the app advertised that way. They advertise those you know, meet other private people who are in your neighborhood and…still, maintaining that confidentiality, you know.

27, African-American, stimulant use less than once a month

Other men were looking for a kind of forum (discussion board) where they can post and respond to HIV-related experiences as a way to learn from each other’s experience:

I think, like, a question answer, like, when you’ve got questions about what’s going on, or with your meds, or your treatment, you know, like, not necessarily a doctor, but kind of like a WebMD, like, how you can post questions or look through other people’s experiences that they may have talked about. Yeah, like, sometimes to go, “Hey is there anybody out there going through this with…Or be like a, look, like, almost a frequently asked questions type of thing where you can go look. Like, type, search symptoms with or side effects with a certain drug and see what people have said. Cause I’ve read in magazines and stuff, oh these are the side effects that you could have, but I like to talk to other people about it.

44, non-Hispanic white, stimulant use less than once a month

#### Resources

Participants mentioned the need to connect to local resources via an app (eg, mental and dental health resources), as well as health education resources that will help them manage their HIV: “I would love local content and that’s I what I think I get from my [local HIV] group, local content…that would keep me going back and then it comes back to the relevancy issue” (41, non-Hispanic white, stimulant use once a week).

#### HIV News/Updates

Some men would like to get information on the latest breakthroughs in HIV medications and related stories that would be relevant or in some way apply to their own life:

I have some news apps and I’ll give you breaking news about different things because I’m a news junkie, but I would appreciate hearing about breakthroughs in HIV or new studies or things like that that are relevant to me that I might want to consider.

54, non-Hispanic white, stimulant use every couple of weeks

Participants also mentioned their desire to find something new and engaging every time they go to their mobile app:

That’s one I have to say about the [local HIV group] in Facebook is that [it has] things to really keep me engaged and every time I go back there I’m like I’m going to find an interesting conversation. I’m going to find an interesting news story. There’s a new event. There’s this constantly something. I know it’s a lot of work but that to me has kept me engaged in its community that I need and what information about.

49, non-Hispanic white, stimulant use every couple of weeks

#### Synching Capabilities

Most participants wanted to see the app synchronized to their other medical records so that it would be easier to keep track of their medical information and have their history readily available during their appointments. They also mentioned the need for synching an adherence app with other computing devices. As noted by one participant, “to have it that you can interface it with your computer. Actually you have the same information on your computer…that will sync up with what you got when you put your password or whatever in” (44, non-Hispanic white, stimulant use less than once a month).

#### Reminders for Health Care

One aspect mentioned by men was the need to get a doctor’s appointment reminder: “being able to integrate it with your provider…your provider’s information. Now there are simple things, you know, to integrate with your calendar so that it reminds when your next doctor’s appointment is. Yeah, simple stuff like that” (46, Hispanic white, stimulant use once a week). Men also indicated the importance of getting a reminder for their medication refill: “Maybe even linking it to your pharmacy so that you could do maybe a refill from that particular app I don’t know if that’s possible, ‘cause I have an app for a refill service and it sends me notifications as when it’s ready” (37, non-Hispanic white, stimulant use almost every day or every day).

Finally, men wanted reminders for managing other aspects of their HIV health care: “Maybe like along the lines of putting in reminders…to send in your program HH [Health and Human Services] paperwork...or information like if there’s something coming up with renewing Ryan White something or something to call your Senator or whatever, maybe” (24, non-Hispanic white, stimulant use once a month).

#### Medication Dose Reminders

Men who frequently struggled to remember to take their medication expressed interest in dose reminders through a mobile app:

I’ve never been med adherent for like the last three years and um, and it's mainly because I always forget. I’m a very forgetful person and um, which is kind of the scary thing but I think like this app thing is kind of cool and I think what would what would be helpful for me if I were to download that app… is like having that notification at the same every day.

28, non-Hispanic white, stimulant use once a week

Some men particularly mentioned a need to get a reminder at times they were most likely to forget:

I think definitely the reminders is probably the biggest piece. Um, and actually tracking for me. So some sort of reminder about taking the medicine and then if you’re you know, maybe over the weekend if you’re a frequent hangout at your friend’s house over the weekend and you’re not home, are you prepared? Did you take your medicine with you when you packed all your essentials? Um, so it would be along those lines.

37, non-Hispanic white, stimulant use almost every day or everyday

However, the need for daily dose reminders was not shared by all men, and some men noted that reminders should include more features than they would normally have in their phones: “at the same time, I think that, like, with the specific idea that you wouldn’t want it to be too simple, cause I’ve already got, like, on my calendar a reminder to take my meds” (24, non-Hispanic white, stimulant use once a month).

#### Graphing and Summarizing

Although some participants did not want to receive reminders for medication doses, they did express interest in being shown a summary of their medication taking over time: “the idea of having to do something every day…does not appeal to me at all, but the benefit of seeing what patterns are in my life over a period of time, I might be willing to commit to doing something and to try that” (54, non-Hispanic white, stimulant use every couple of weeks).

Men noted that such a feature would help them keep an accurate record of the number of days they have missed their medication and bring that up to their doctor’s appointment: “The second thing is it would be nice if, at the end of the month, it would tell me how many doses I missed...Because some of the, you know, things that I do, like checkups with my doctor, they want to know that” (35, non-Hispanic white, stimulant use once a month).

Other participants wanted this feature so that they could use the information as motivation to adhere more consistently to their medication:

I wanna see what my numbers are for that month. So…the next time I go to the doctor, let’s say I missed two weeks for whatever reason, which I have...I can see that my numbers went down just so that I can put in my numbers, you know, into the calendar when do I get my checkups...so that I have a visual to say, you know, these people aren’t full of shit, that it really does matter, or something like that.

46, non-Hispanic white, stimulant use once a month

The quote above reflects this participant’s belief that logging periods of ART non-adherence could motivate him to be more adherent by providing clear evidence that missed doses directly affects his health during subsequent visits to his health care provider.

#### Adherence Performance Feedback

Participants wanted to receive a message that will positively reinforce their medication adherence: “every time I look down at the app, if there was something that spoke to a statistic, a study statistic about the power of adherence…because to me, in addition to the check-in, if I saw that and I hadn’t taken them yet, that might motivate me to say ‘You know what?’” (49, non-Hispanic white, stimulant use every couple of weeks).

Men noted that feedback should avoid being presented in a parental or shaming manner, while some men thought that using humor to provide feedback about missed doses would be effective*:* “if you’re gonna do motivational, positive messages...you should say ‘Hey, idiot, you forgot your medicine.’ You know, which makes it hilarious for me” (46, non-Hispanic white, stimulant use almost every day or every day).

#### Health File

Overall, men expressed their need to have a file where they can store all their health-related information. As one participant noted, “So it is nice to have one location where all your medication and stuff is, if you need it in an emergency situation” (54, non-Hispanic white, stimulant use every couple of weeks).

## Discussion

### Principal Results

The successful dissemination and uptake of mobile phone app interventions addressing the HIV prevention and treatment continuum of care requires that persons are willing to download and sustain use of the app, as well as providing content and features that are culturally relevant for the target population. In this study, we explored the features and functions of mobile phone apps that stimulant-using HIV-positive MSM considered when deciding to download and sustain their use of apps in general over time, as well as specific features and functions that they would like to see incorporated into an app to help them manage ART adherence more effectively.

Men who participated in these focus groups appear to already be using a variety of mobile phone apps to help them manage HIV and substance use. When asked about apps they currently had on their mobile phones, participants stated that they had apps to help them navigate their health care (eg, clinic, insurance, and pharmacy apps), medications, and HIV. In addition, at least 1 participant noted that he used an app to track the number of days of sobriety. These results suggest that apps designed for stimulant-using HIV-positive MSM will need to fill a unique niche between currently available apps for HIV and apps addressing substance use. To our knowledge, there are no current apps that specifically address the intersection of HIV and stimulant use. Given that stimulant-using HIV-positive men are at high risk for ART adherence problems [5,6,11], developing an app that incorporates the lessons learned in this study is warranted.

There were three features specific to men’s decisions to download an app to their phone. Men believed apps should be either free or very low cost. However, men scrutinized apps with some cost associated more so, by reading reviews or asking for suggestions for their friends. In contrast, men stated that they were inclined to download a free app to their phone that they knew little about since there was no cost involved and they could easily remove it from their phone later. Recommendations from friends appear to be an important motivator to men in this study, with respect to downloading an app. HIV app developers should consider ways that stimulant-using HIV-positive MSM can easily recommend an app to friends as an avenue to disseminate the app broadly, such as including a way to send an email or text to friends with the app information and downloading instructions.

Perhaps the greatest challenge to ensuring the dissemination and longevity of HIV prevention and treatment apps is to understand the features and functions that will motivate men to sustain their use of these apps over time. Stimulant-using HIV-positive MSM in this study noted several features and functions that were associated with downloading *and* sustained app use, including having control over most features of the app, how useful and engaging it was, its security features, and the credibility of the app. A prior study of HIV-negative men’s preferences for an HIV prevention app found similar sentiments [21]. These are common components of technology adoption models [26] and many of these dimensions —especially creating interventions that are engaging and useful—are common “best practices” across most technology platforms [30]. We found it noteworthy that some men in this study appeared to express little concern about security of apps on their mobile phone. This is, in part, likely a self-selection bias that men who are willing to participate in focus groups are more open about sensitive personal information, such as their HIV status. However, this finding also suggests that app developers should weigh the costs and benefits of each security feature of the app to ensure that these features are not overly burdensome, since low burden is an influential factor for adoption and use of technologies [31]. Finding the optimal balance of security, engagement, usefulness, control capabilities, and credibility will be critical to sustained use of HIV treatment apps.

There were a number of different features and functionality preferences stimulant-using HIV-positive MSM would like to see incorporated in an ART adherence mobile phone app, many of which reflected the preferences of HIV-positive men and women in a prior study by Schnall et al [22]. Men in the current study expressed a desire to connect with outside local and social network resources through the app, including socializing with other HIV-positive men, connecting to local mental health and other resources, viewing breaking news about HIV, and connecting to their medical chart. Although connecting to external resources and health records undoubtedly would be helpful for persons with HIV, there are significant barriers to achieving some of these recommendations. For example, integrating an ART adherence app with commercial apps (eg, dating and sex-seeking apps such as Grindr, Scruff, and Growlr) or electronic health records requires agreement from, and coordination with, the leadership and developers of those outside organizations (such as developers of health care apps, including MyChart or Kaiser Permanente). A platform for building relationships between researchers wishing to test HIV prevention and treatment apps and existing health and for-profit companies is needed to advance cooperation between these key stakeholders.

Other app features and functionality preferences that men noted were specific to ART adherence self-monitoring, including whether receiving medication dose reminders was acceptable, what information about their adherence performance would be helpful and how to frame feedback in response to missed doses. Overall, although there was some concern about monitoring each dose of medication, men were in favor of seeing a summary of their ART adherence performance over time. They stated that receiving such feedback may motivate them to be more adherent by directly seeing a summary of their performance over time. In addition, they could use the summary during appointments with health care providers to communicate more accurate estimates of their ART adherence. ART adherence app researchers may want to include explicit benefits of daily dose monitoring to participants using the app to increase “buy-in” to self-monitor medication doses. Finally, men expressed the desire for feedback about missed doses to be framed in a positive, non-authoritative, and possibly humorous way. These same qualities about the language and tone of successful apps were reflected by HIV-negative MSM in a recent study [21].

### Limitations

There are some important limitations to this study. First, the results from these focus groups are not intended to be generalizable to all stimulant-using HIV-positive MSM, persons with HIV, or MSM in general in the United States. Most men in this study were non-Hispanic white, which does not reflect the racial and ethnic distribution of HIV in the United States [1]. Thus, future research should assess mobile phone app preferences among a more diverse sample of MSM to determine whether similar attitudes are expressed. In addition, we recruited men residing in or near only two metropolitan areas, San Francisco and Minneapolis. Therefore, these results may not reflect men living in other areas of the United States or more rural regions. These results are meant to be first steps in more fully understanding the needs of stimulant-using HIV-positive MSM with respect to mobile phone apps to address ART adherence.

### Conclusions

Despite the limitations of this study, we were able to recruit a relatively high proportion (42%) of participants reporting weekly or more stimulant use. Advancing novel ART adherence interventions for these men are particularly necessary given that weekly or more frequent stimulant use has been found to be associated with intermittent ART utilization and elevated viral load [32]. The results from this study suggest critical design elements that may need to be considered during development of ART adherence-related mobile phone apps for this, and possibly other, high-risk groups.

## Abbreviations

AIDS: acquired immune deficiency syndrome
ART: antiretroviral therapy
HIV: human immunodeficiency syndrome
MSM: men who have sex with men
TAM: Technology Adoption Model
